# Draft Genome Sequence of a Natural Root Isolate, *Bacillus subtilis* UD1022, a Potential Plant Growth-Promoting Biocontrol Agent

**DOI:** 10.1128/genomeA.00696-15

**Published:** 2015-07-09

**Authors:** Usha Bishnoi, Shawn W. Polson, D. Janine Sherrier, Harsh P. Bais

**Affiliations:** a Department of Plant and Soil Sciences, University of Delaware, Newark, Delaware, USA; b Delaware Biotechnology Institute, Newark, Delaware, USA; c Center for Bioinformatics and Computational Biology, University of Delaware, Newark, Delaware, USA

## Abstract

*Bacillus subtilis*, which belongs to the phylum *Firmicutes*, is the most widely studied Gram-positive model organism. It is found in a wide variety of environments and is particularly abundant in soils and in the gastrointestinal tracts of ruminants and humans. Here, we present the complete genome sequence of the newly described *B. subtilis* strain UD1022. The UD1022 genome consists of a 4.025-Mbp chromosome, and other major findings from our analysis will provide insights into the genomic basis of it being a plant growth-promoting rhizobacterium (PGPR) with biocontrol potential.

## GENOME ANNOUNCEMENT

*Bacillus subtilis* is arguably one of the most widely studied plant growth-promoting Gram-positive bacteria, having a great impact on agriculture, although information pertaining to its ecological and plant interactions is relatively unknown. Our published work has revealed that supplementation of *B. subtilis* strain UD1022 induces plant defense and growth promotion in various plant systems (1–5). Previous studies indicated that even though various isolates of *B. subtilis* revealed 98% sequence identity at the 16S rRNA level, the DNA-DNA reassociation analysis showed only 58% relatedness among the various *B. subtilis* strains (6). These observations encouraged us to decipher the UD1022 genome sequence, and the analysis of the genome will provide insight into the mechanisms underlying its nature as a plant growth-promoting rhizobacterium (PGPR) and its biocontrol potential. Its plant growth-promoting and biocontrol activities provide a green alternative to the synthetic molecules commonly used for crop yield and disease protection in the present agricultural system (7).

The *B. subtilis* complex represents an assembly of closely related species, and phylogenetic analysis of the 16S rRNA gene fails to differentiate species within the complex due to the highly conserved nature of the gene. Here, we used the PacBio approach (8, 9) to analyze the UD1022 genome. Genomic DNA of UD1022 was extracted using a Qiagen DNA isolation kit. The genomic DNA was randomly sheared to ~10-kb target size using G-tubes (Covaris, Inc.). Poly(dA) tails were added to the 3′ ends using terminal deoxynucleotidyl transferase (TDT). The poly(dA)-tailed library was then annealed with poly(DT) sequencing primer and sequenced using a DNA/polymerase binding kit 2.0 (C2/C2 chemistry) with a MagBead loading kit and 120-min sequencing time using six single-molecule real-time (SMRT) cells (3 × 2-kb insert and 3 × 10-kb insert) on a Pacific Biosciences RS II sequencer. The resulting mean subread length was 3,834 bp, and the *N*_50_ length was 5,669 bp. The HGAP protocol implemented in SMRT Analysis version 2.0.1 was used to assemble the UD1022 genome (10). This resulted in two contigs in the HGAP output of 63,171 bp and 3,967,258 bp, with 188.77× mean coverage. The manual finishing process resulted in one 4,025,326-kb circular chromosome, with 43.89% average G+C content. Final assembly was polished using the Quiver consensus algorithm included in the SMRT Analysis software package. Base modifications were identified using the base modification analysis protocol (Pacific Biosciences). Genes were predicted using the RAST server (11) and by the NCBI GenBank Prokaryotic Genome Annotation Pipeline (PGAP). PGAP located 4,129 features, including 3,933 coding sequences (CDSs), 79 pseudogenes, 10 rRNA operons, 86 tRNA genes, and 1 noncoding RNA (ncRNA) (ribozyme: RNase P). Compared to the genome sequence of *B. subtilis* strain 168 (accession no. NC_000964.3), 3,583 CDSs had a strong bidirectional homolog (>85% identity), 164 had weaker or unidirectional homologs (>50% identity), 439 were unique to UD1022, and 421 were unique to *B. subtilis* strain 168. CDSs unique to UD1022 included genes for various amino acid biosynthesis, transport, and metabolism, cell wall proteins, carbohydrate metabolisms, and metal (copper, cobalt-zinc-cadmium) and antibiotic (β-lactam, tetracycline, and vancomycin) resistance.

### Nucleotide sequence accession numbers.

All raw and assembled data for the project have been submitted to NCBI under BioProject no. PRJNA284309, with the complete genome sequence deposited in GenBank under the accession no. CP011534. The UD1022 strain is deposited at ATCC with the accession no. PTA11857.
